# Calorie restriction has no effect on bone marrow tumour burden in a Vk*MYC transplant model of multiple myeloma

**DOI:** 10.1038/s41598-022-17403-9

**Published:** 2022-07-30

**Authors:** Alanah L. Bradey, Stephen Fitter, Jvaughn Duggan, Vicki Wilczek, Connor M. D. Williams, Emma AJ. Cheney, Jacqueline E. Noll, Pawanrat Tangseefa, Vasilios Panagopoulos, Andrew C. W. Zannettino

**Affiliations:** 1 Myeloma Research Laboratory, Faculty of Health and Medical Sciences, School of Biomedicine, University of Adelaide, Adelaide, Australia; 2 Precision Cancer Medicine Theme, Solid Tumour Program, South Australian Health and Medical Research Institute, Adelaide, Australia; 3 Department of Haematology, Royal Adelaide Hospital, Adelaide, Australia; 4 Central Adelaide Local Health Network, Adelaide, Australia

**Keywords:** Bone cancer, Cancer microenvironment, Myeloma, Cancer prevention, Cancer metabolism, Risk factors, Experimental models of disease, Preclinical research

## Abstract

Multiple myeloma (MM) is an incurable haematological malignancy, caused by the uncontrolled proliferation of plasma cells within the bone marrow (BM). Obesity is a known risk factor for MM, however, few studies have investigated the potential of dietary intervention to prevent MM progression. Calorie restriction (CR) is associated with many health benefits including reduced cancer incidence and progression. To investigate if CR could reduce MM progression, dietary regimes [30% CR, normal chow diet (NCD), or high fat diet (HFD)] were initiated in C57BL/6J mice. Diet-induced changes were assessed, followed by inoculation of mice with Vk*MYC MM cells (Vk14451-GFP) at 16 weeks of age. Tumour progression was monitored by serum paraprotein, and at endpoint, BM and splenic tumour burden was analysed by flow cytometry. 30% CR promoted weight loss, improved glucose tolerance, increased BM adiposity and elevated serum adiponectin compared to NCD-fed mice. Despite these metabolic changes, CR had no significant effect on serum paraprotein levels. Furthermore, endpoint analysis found that dietary changes were insufficient to affect BM tumour burden, however, HFD resulted in an average two-fold increase in splenic tumour burden. Overall, these findings suggest diet-induced BM changes may not be key drivers of MM progression in the Vk14451-GFP transplant model of myeloma.

## Introduction

Multiple myeloma (MM) is a fatal haematological malignancy characterised by the uncontrolled proliferation of plasma cells (PC) within the bone marrow (BM). Each year over 160,000 new cases of MM are diagnosed worldwide, accounting for approximately 10% of all haematological malignancies^1^. MM is universally preceded by an indolent, asymptomatic disease known as monoclonal gammopathy of undetermined significance (MGUS). MGUS is highly prevalent, affecting approximately 3% of the population over the age of 50 years, with approximately 1% of MGUS patients progressing to MM each year^2–4^. Presently, no therapeutic interventions are available for MGUS patients, with treatment only initiated in active MM disease, when patients display often irreversible end organ damage^5^. Globally, MM has a poor 5-year survival rate of between 9 and 64%^1^. To improve upon current survival rates it is imperative to understand the mechanisms by which disease progresses from asymptomatic MGUS to malignant MM. Understanding the factors underlying progression may enable the induction of early intervention strategies for the preventative treatment of MGUS patients.

Obesity is associated with many pathological conditions and has been identified as a risk factor for multiple cancer types^6,7^. A number of retrospective studies have identified obesity as a significant risk factor for the progression of MGUS to MM^8,9^. Furthermore, a meta-analysis combining data from more than 1.5 million patients found high BMI and waist circumference were positively associated with increased MM mortality^10^. The increased risk of MM with obesity is thought to be due to changes in the BM microenvironment (BMME) that are associated with increased nutrient availability. Importantly, whilst the progression of myeloma has been classically viewed as a multi-step process resulting from the accumulation of genetic mutations^11–13^, recent data has highlighted the importance of extrinsic signals from cellular and non-cellular components of the BM microenvironment. Indeed, DNA barcoding of genetically identical MM PCs revealed the proliferative fate of cells was entirely dependent on the BMME in which they reside^14^. Moreover, studies using transgenic mice engineered to express MM growth factors revealed that MGUS PCs had the same proliferative potential as MM PCs when provided with a growth supportive BMME^15^. In line with these findings, a study by Lwin et al. found that diet-induced obesity was sufficient to create a permissive environment, enabling the growth of 5TGM1 PCs in the usually non-permissive C57BL/6J mouse strain^16^.

Despite obesity being an established risk factor for MM, few studies have investigated the potential of dietary intervention to prevent disease progression. In many animal models, calorie restriction (CR), without micronutrient deficiency, has shown to extend lifespan and improve healthspan by delaying the onset of age-related diseases^17^. Preclinical models of several solid cancers have shown that CR can delay tumour onset and progression^18–20^. Furthermore, a longitudinal study in rhesus monkeys showed a reduction in cancer incidence with CR feeding^21^. The mechanisms underlying the antitumorigenic effects of CR are unclear, however, studies suggest that it is, at least in part, due to alterations in systemic metabolic and inflammatory signalling pathways. Notably, CR is associated with a reduction in serum glucose levels and growth factors such as insulin and insulin-like growth factor (IGF-1)^22,23^, both of which have been identified as MM PC growth factors^24,25^. Moreover, CR is associated with changes to metabolic adipokine signalling due to alterations in the amount and function of adipose tissue. Weight loss and a reduction of white adipose tissue (WAT) are characteristic of CR. The reduction in WAT leads to reduced circulating levels of WAT-secreted factors such as the pro-inflammatory adipokine leptin, high levels of which has been associated with occurrence and development of MM^26^.

In addition to whole-body metabolic changes, CR leads to alterations in BM composition. Specifically, in mice, a 30% reduction in daily food intake (30% CR) leads to an expansion of BM adipose tissue (BMAT) and a reduction in bone mass^27^. This contrasts with decreased WAT under CR, highlighting the phenotypically distinct nature of adipose tissue depots. BMAT is now recognised as an endocrine organ that secretes a large range of cytokines and adipokines which play important roles in both local BM niche function and systemic metabolism^28^. Under conditions of energy deficiency, such as 30% CR, expanded BMAT becomes a major source of the anti-inflammatory adipokine adiponectin, resulting in increased circulating serum adiponectin levels^29^. Adiponectin enhances insulin sensitivity, leading to improved glucose homeostasis^30^. In MM, low serum adiponectin levels are a biomarker for progression of MGUS to MM^31^. Moreover, mechanistic studies have shown that adiponectin plays an anti-tumour role, with adiponectin-deficient mice displaying greater myeloma tumour growth^32^.

Given the anti-tumourigenic changes associated with CR, we hypothesised that CR would create a non-permissive microenvironment leading to delayed MM progression. Herein, we investigated this hypothesis using an immune-competent transplant Vk*MYC (Vk14451-GFP) mouse model of MM, which accurately recapitulates many of the features of human disease^33^. Prior to initiation of the Vk*MYC model, C57BL/6J mice were fed dietary regimes [30% CR diet, normal chow diet (NCD), or high fat diet (HFD)] to establish metabolic and BMME profiles. Our study revealed that diet-induced metabolic and microenvironmental changes were insufficient to impact BM tumour burden in the Vk*MYC pre-clinical model of myeloma.

## Results

### Dietary intervention altered body weight, composition and glucose tolerance

**Figure 1:**
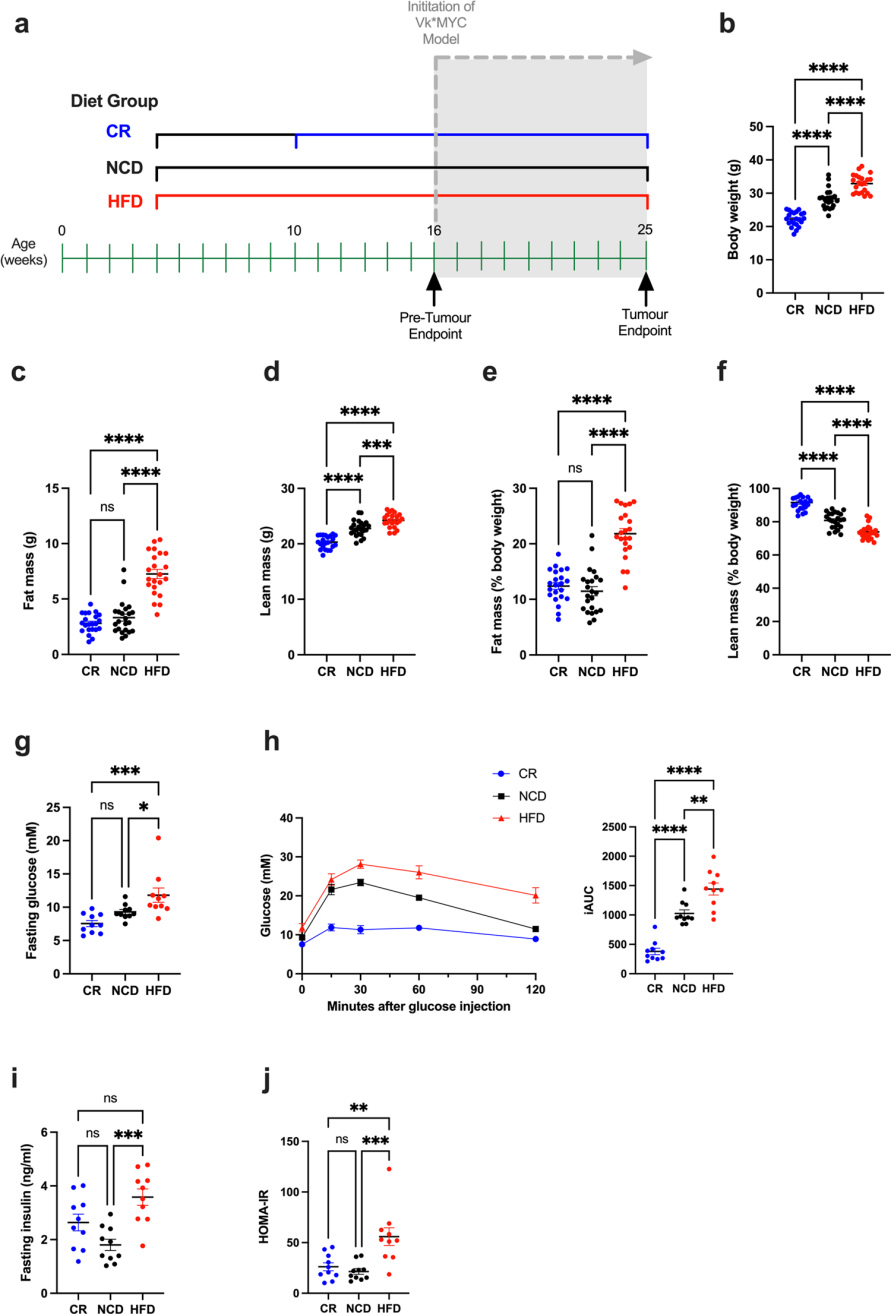
Effects of dietary regimens on body weight, composition and glucose tolerance at pre-tumour endpoint. (**a**) Experimental timeline showing duration of diets and analysis endpoints, (**b**) Body weight, (**c**) Fat mass, (**d**) Lean mass, (**e**) Fat mass (% body weight), (**f**) Lean mass (% body weight), (**g**) Fasting blood glucose levels, (**h**) Glucose clearance over time, (**i**) Fasting insulin levels, (**j**) HOMA-IR scores. Error bars SEM, (**b**–**f**) data n = 22–23/group, (**g**–**k**) data n = 10/group, ns p > 0.05, *p < 0.05, **p < 0.01, ***p < 0.001, ****p < 0.0001, ordinary one-way ANOVA with Tukey’s multiple comparisons test.

To ensure that dietary protocols in this study created the anticipated body compositional and metabolic changes, a cohort of mice (16 weeks of age; pre-tumour endpoint Fig. 1a) were analysed prior to tumour initiation. As expected, CR mice had significantly reduced body weight compared to NCD and HFD mice, while HFD mice had significantly increased body weight compared with NCD and CR mice (Fig. 1b). Body composition analysis revealed that the main contributor to HFD-fed mice weight gain was increased total fat (Fig. 1c), whilst in CR-fed mice, weight loss was mainly attributed to a decrease in lean mass (Fig. 1d). Surprisingly we did not observe decreased fat (Fig. 1c), or fat mass relative to body weight (Fig. 1e) in CR-fed mice relative to NCD-fed mice. Despite this, CR-fed mice had significantly increased lean mass relative to body weight compared to both NCD- and HFD-fed mice (Fig. 1f).

In addition to the analysis of body weight and tissue-compositional changes, glucose tolerance testing was performed prior to tumour inoculation. HFD-fed mice had significantly elevated fasting blood glucose levels compared to both NCD and CR diet fed mice (Fig. 1g), as well as reduced glucose clearance (Fig. 1h). In contrast, CR-fed mice displayed a trend towards lower fasting glucose levels, relative to NCD and a marked improvement in glucose tolerance as evidenced by very rapid glucose clearance (Fig. 1g,h). Further analysis revealed significant elevation in fasting blood insulin levels in HFD mice compared to NCD control mice (Fig. 1i). Taken together these results indicated a diet-induced Type 2 diabetes mellitus (T2DM)-like phenotype in HFD-fed mice, as calculated by significantly higher homeostatic model assessment of insulin resistance (HOMA-IR) scores (Fig. 1j).

### Dietary intervention altered the BMME and serum adipokine levels

**Figure 2:**
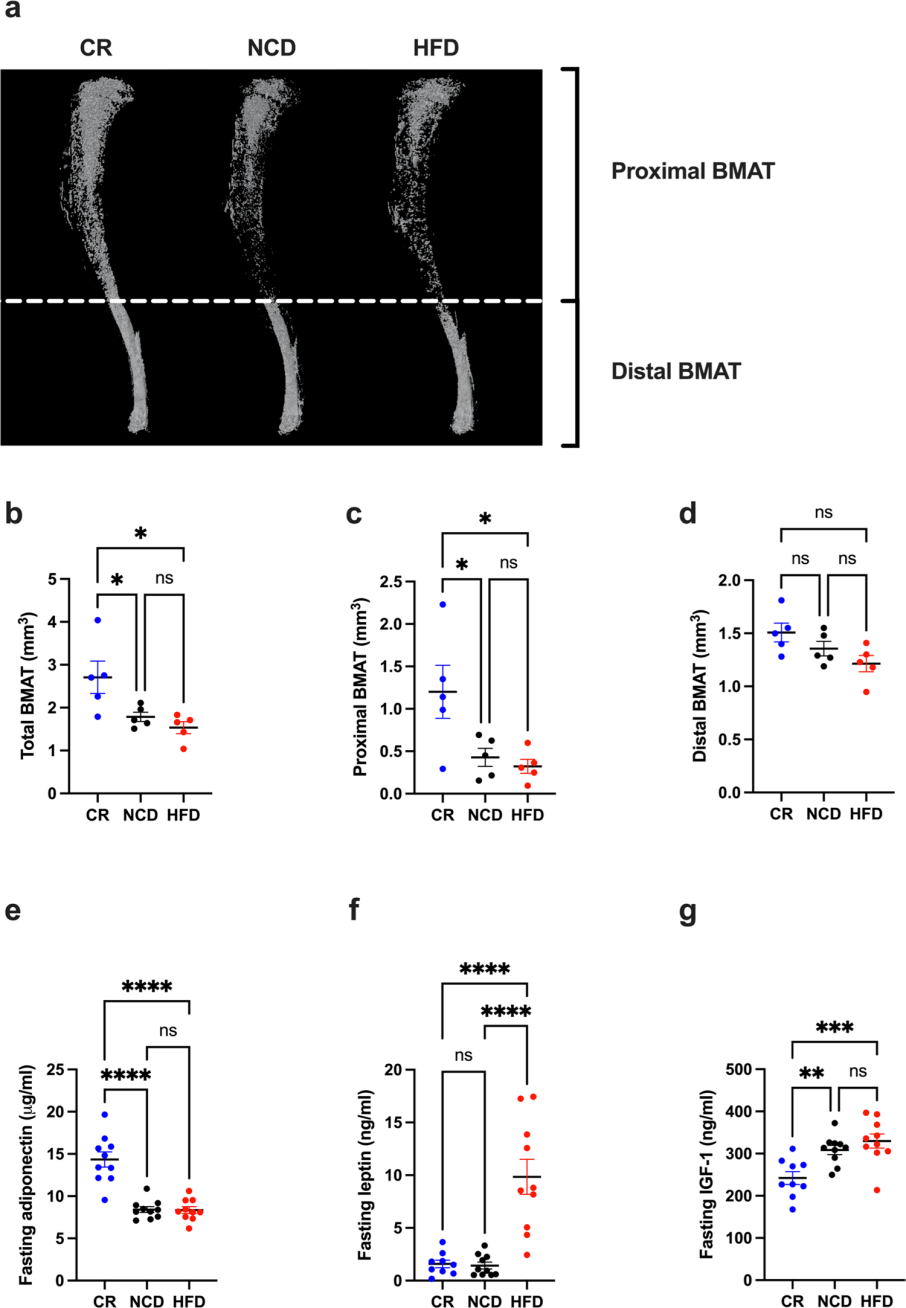
CR promotes proximal BM adiposity and an increase in serum adiponectin levels. (**a**) Representative images of osmium tetroxide stained tibias, (**b**) Total BMAT, (**c**) Proximal BMAT, (**d**) Distal BMAT, (**e**) Fasting serum adiponectin levels, (**f**) Fasting serum leptin levels, (**g**) Fasting serum IGF-1 levels. Error bars SEM, (**a**–**d**) n = 5/group, (**e**–**g**) n = 10/group, ns p > 0.05, *p < 0.05, **p < 0.01, ***p < 0.001, ****p < 0.0001, Ordinary one-way ANOVA with Tukey’s multiple comparisons test.

Given the importance of the BMME in myeloma progression, diet-induced changes to bone micro-architecture and composition were characterised in our pre-tumour endpoint mice at 16 weeks of age using μCT for analysis of trabecular and cortical bone, followed by osmium tetroxide staining for quantification of BM adiposity. Whilst there was no significant difference in trabecular bone parameters in CR mice compared to NCD, there was a significant decrease in cortical bone in the proximal tibia (Table 1). Moreover, analysis of osmium tetroxide-stained tibias revealed a significant increase in BMAT, specifically proximal BMAT, with CR compared to NCD and HFD (Fig. 2a–d). In line with BMAT being a major source of adiponectin under CR, CR mice had a significant increase in serum adiponectin compared to NCD and HFD mice (Fig. 2e). Serum leptin levels in CR mice were not significantly reduced compared to NCD controls (Fig. 2f). Analysis of IGF-1 levels revealed a significant reduction in serum IGF-1 in CR-fed mice compared to both NCD- and HFD-fed mice (Fig. 2g). In contrast to CR and NCD mice, HFD-fed mice displayed no changes in cortical bone but a significant decrease in trabecular thickness (Table 1). Notably, 12-weeks on a HFD was insufficient to alter BMAT quantity or serum adiponectin levels relative to NCD-fed mice (Fig. 2a,e), however, HFD-fed mice had significantly higher serum leptin levels relative to CR- and NCD-fed mice (Fig. 2f).

**Table 1:** The effect of dietary regimens on trabecular and cortical bone micro-architecture.

Bone properties	CR	NCD	HFD
**Proximal tibia**			
Trabecular bone			
BV/TV (%)	4.42 ± 0.48	4.85 ± 0.45	4.65 ± 0.155
Tb.Th (mm)	0.0451 ± 0.00061	0.0451 ± 0.00063	0.0429 ± 0.00033*^#^
Tb.N (1/mm)	0.98 ± 0.11	1.08 ± 0.10	1.09 ± 0.04
Tb.Sp (mm)	0.68 ± 0.04	0.62 ± 0.02	0.69 ± 0.04
Cortical bone			
Tt.Ar (mm^2^)	2.94 ± 0.092	2.92 ± 0.079	3.09 ± 0.088
Ct.Ar (mm^2^)	0.81 ± 0.025*	0.88 ± 0.013	0.87 ± 0.010
Ct.Ar/Tt.Ar (%)	27.44 ± 0.784*	30.14 ± 0.694	28.13 ± 0.554
**Midshaft tibia**			
Cortical bone			
Tt.Ar (mm^2^)	1.429 ± 0.04	1.484 ± 0.05	1.449 ± 0.03
Ct.Ar (mm^2^)	0.835 ± 0.02	0.880 ± 0.03	0.867 ± 0.01
Ct.Ar/Tt.Ar (%)	58.48 ± 0.40	59.34 ± 0.75	59.87 ± 0.54

Data are expressed as mean ± SEM from n = 5 mice/group; ordinary one-way ANOVA with Tukey’s multiple comparisons test. *BV/TV* bone volume/total volume, *Tb.Th* trabecular thickness, *Tb.N* trabecular number, *Tb.Sp* trabecular separation, *Tt.Ar* total cross-sectional area inside the periosteal envelope, *Ct.Ar* cortical bone area, *Ct.Ar*/*Tt.Ar* cortical area fraction. *p < 0.05 versus NCD group. ^#^p < 0.01 compared to CR group.

In addition to the analysis of adipokine levels, inflammatory changes to the BMME were also investigated prior to tumour initiation using gene expression analysis of inflammatory markers. In line with the pro-inflammatory nature of HFD-induced obesity, we observed a significant increase in gene expression of *TNFα* in the tibias of HFD-fed mice relative to CR- and NC-fed mice (Supp Fig. 1). Expression levels of *IL1β*, *IL6* and *MPO* showed a similar trend but failed to reach significance (Supp Fig. 1).

### Diet-induced changes to haematological parameters

**Figure 3:**
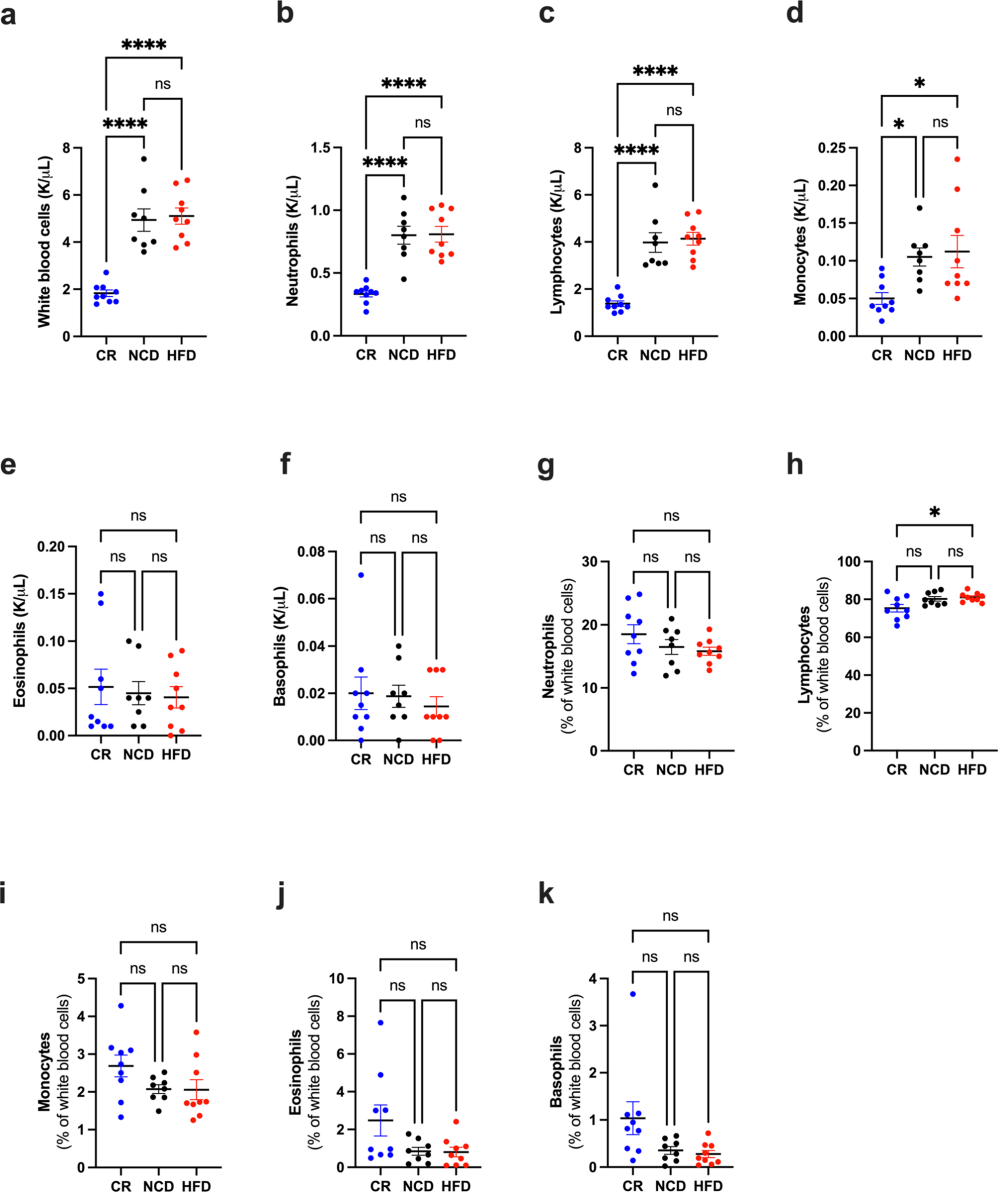
Peripheral blood cell counts at pre-tumour endpoint. (**a**) White blood cell count, (**b**) Neutrophil count, (**c**) Lymphocyte count, (**d**) Monocyte count, (**e**) Eosinophil count, (**f**) Basophil count, (**g**) % Neutrophils, (**h**) % Lymphocytes, (**i**) % Monocytes, (**j**) % Eosinophils, (**k**) % Basophils. Error bars SEM, n = 8–9/group, ns p > 0.05, *p < 0.05, **p < 0.01, ***p < 0.001, ****p < 0.0001.

In addition to examining diet-induced metabolic and BM microenvironmental changes, we investigated the effect of diet on normal haematopoiesis prior to tumour initiation. We observed a significant decrease in total white blood cells, lymphocytes, neutrophils and monocytes with CR relative to both NCD- and HFD-fed mice (Fig. 3a–f), however the proportion of cell subsets remained unchanged (Fig. 3g–k). No differences in blood cell sub-types were observed in HFD-fed mice relative to NCD-fed mice (Fig. 3a–f).

### Diet-induced metabolic and BMME changes do not reduce myeloma tumour burden in the BM

**Figure 4:**
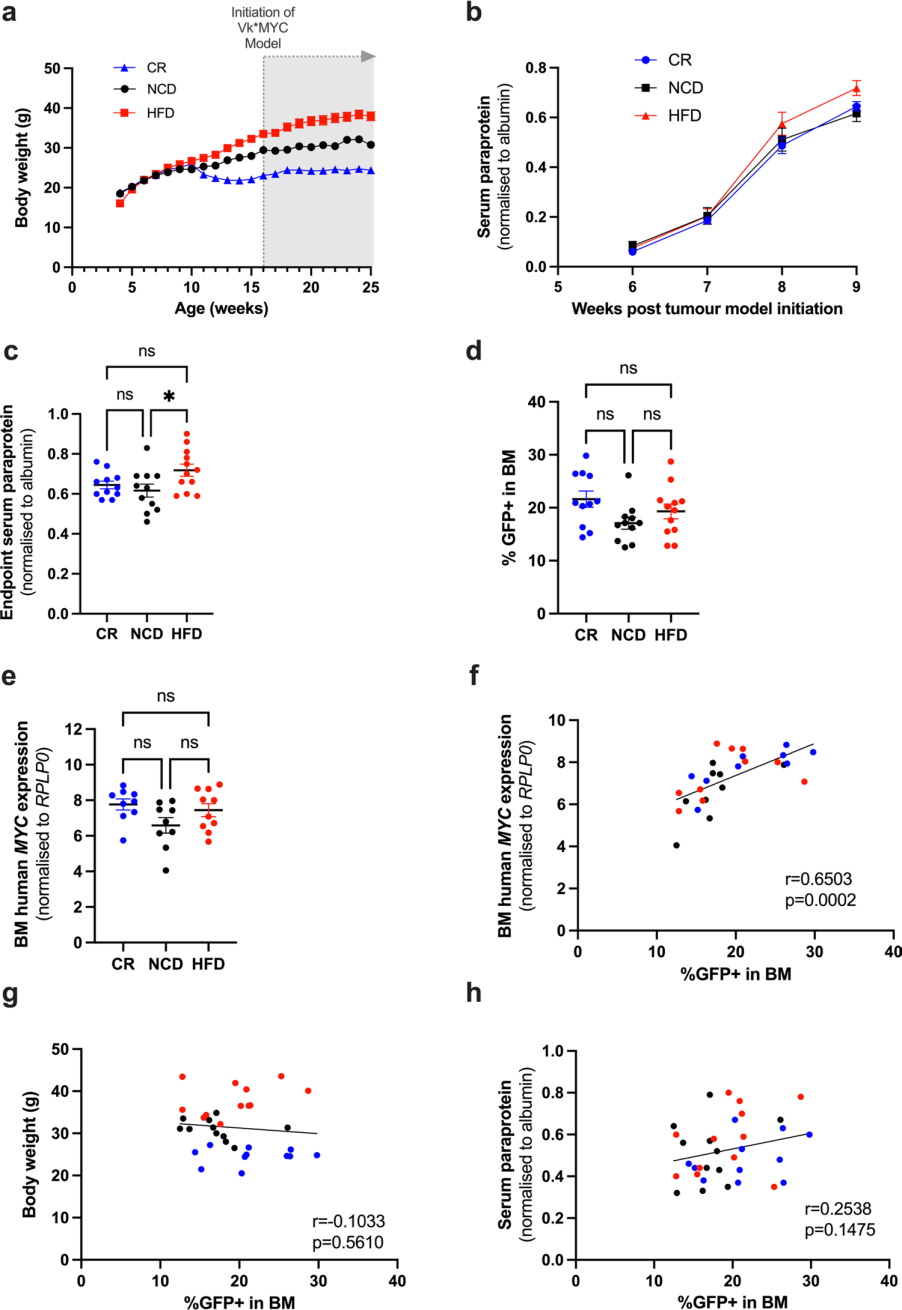
CR and HFD-feeding have no effect BM tumour burden. (**a**) Body weight over time, (**b**) Serum paraprotein levels over time, (**c**) Serum paraprotein levels at tumour endpoint (9 weeks post tumour initiation), (**d**) %GFP+ in the BM, (**e**) BM human *MYC* expression, (**f**) Correlation between %GFP+ in BM and BM human *MYC* expression, (**g**) Correlation between body weight and %GFP+ in BM, (**h**) Correlation between serum paraprotein and %GFP+ in BM. Error bars SEM, (**a**–**e**) n = 11–12 mice/group, ordinary one-way ANOVA with Tukey’s multiple comparisons test**,** (**f**–**h**) Combined data (n = 34 mice) from n = 11–12 mice/group, dot colours corresponds to diet group, blue dots: CR, black dots: NCD, red dots: HFD, correlation analysis, ns p > 0.05, *p < 0.05, **p < 0.01, ***p < 0.001, ****p < 0.0001.

To investigate the effects of diet-induced metabolic and microenvironmental changes on MM development and progression, a cohort of mice were injected with GFP+ Vk*MYC PCs via the tail vein at 16 weeks of age following dietary intervention (see schematic in Fig. 1a). All mice were maintained on their assigned diet for the duration of the study, with weight changes monitored over time (Fig. 4a). Tumour burden was monitored over the course of the experiment using serum paraprotein electrophoresis (SPEP). At 6-weeks post tumour inoculation, distinct paraprotein bands could be visualised for quantification (Supp Fig. 2), with tumour burden increasing exponentially from 7–8 weeks post inoculation (Fig. 4b). At tumour endpoint (9 weeks post tumour injection), paraprotein analysis revealed a significant increase in whole body tumour burden in HFD-fed mice compared to NCD, but no significant difference in CR-fed mice compared to NCD or HFD (Fig. 4c). To investigate differences in BM tumour burden, GFP+ tumour cells in the hind limbs were enumerated using flow cytometry. No significant differences in %GFP+ tumour cells in the BM were observed between diet groups (Fig. 4d). qPCR was performed as an independent measure of tumour burden using tumour-specific primers designed to amplify human *MYC*. Gene expression analysis validated our findings, revealing no significant difference in human *MYC* expression between diet groups (Fig. 4e), and a strong correlation with %GFP+ tumour cells in the BM (Fig. 4f). Moreover, body weight was not a determinant of BM tumour growth, with no significant correlation between %GFP tumour cells and body weight irrespective of diet group (Fig. 4g). Next, we performed a correlation analysis to interrogate the discrepancy between the SPEP data and BM tumour burden. No significant correlation between serum paraprotein levels and %GFP+ tumour cells in the BM was observed (Fig. 4h) suggesting that the elevation in serum paraprotein levels in HFD-fed mice was independent of tumour growth in the BM.

### HFD promotes MM tumour growth in the spleen

**Figure 5:**
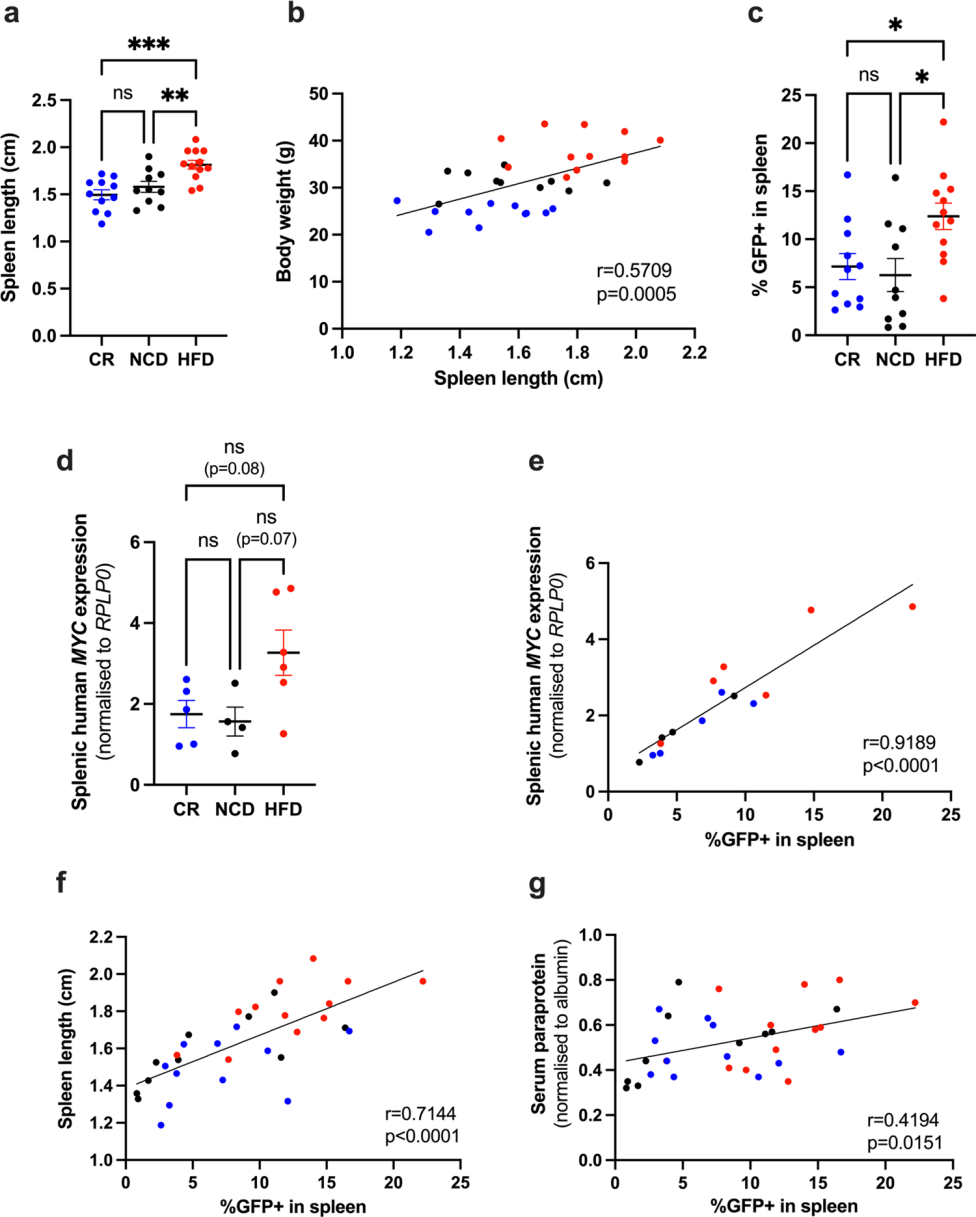
HFD promotes MM tumour growth in the spleen. (**a**) Spleen length, (**b**) Correlation between spleen length and body weight, (**c**) %GFP+ in spleen, (**d**) Splenic human *MYC* expression, (**e**) Correlation between %GFP+ in spleen and splenic human *MYC* expression, (**f**) Correlation between spleen length and %GFP+ in spleen, (**g**) Correlation between %GFP+ in spleen and serum paraprotein. Error bars SEM, (**a**,**c**) n = 10–12 mice/group, (**d**) n = 4–6 mice/group, Ordinary one-way ANOVA with Tukey’s multiple comparisons test, (**e**) Combined data (n = 15 mice) from n = 4–6 mice/group, (**f**,**g**) Combined data (n = 33 mice) from n = 10–12 mice/group, dot colours corresponds to diet group, blue dots: CR, black dots: NCD, red dots: HFD, correlation analysis, ns p > 0.05, *p < 0.05, **p < 0.01, ***p < 0.001, ****p < 0.0001.

In mice, the spleen is a major site of extramedullary haematopoiesis and a frequent site of myeloma tumour growth^33–35^. To investigate differences in extramedullary tumour burden between diets, spleens were excised at experimental endpoint for morphological and cytometric analysis. No obvious extramedullary tumour lesions could be observed in spleens (data not shown), however assessment of spleen length at tumour endpoint revealed spleens from HFD-fed mice were significantly larger than NCD and CR-fed mice (Fig. 5a), with spleen length correlating with body weight (Fig. 5b). Notably, prior to tumour initiation, spleens of HFD-fed mice were significantly heavier than CR-fed mice (Supp Fig. 3). These data suggest that splenomegaly at tumour endpoint was promoted by HFD-induced obesity. Analysis of splenic tumour burden by flow cytometry revealed an average two-fold increase in %GFP+ tumour cells in HFD-fed mice compared to CR-fed and NCD-fed controls (Fig. 5c). These findings were further supported by qPCR which revealed an increase in human *MYC* gene expression in spleens from HFD-fed mice, and a strong correlation between splenic %GFP+ tumour cells and human *MYC* gene expression in HFD-fed mice (Fig. 5d,e). Of note, splenic tumour burden, as assessed by flow cytometry, was correlated with spleen length (Fig. 5f) and serum paraprotein levels (Fig. 5g), suggesting splenic tumour growth is a major contributor of whole-body tumour burden in the Vk*MYC model.

### Comparison of adipokine levels at pre-tumour and tumour endpoints

At tumour endpoint, serum levels of adiponectin and leptin were assessed by ELISA and compared to pre-tumour endpoint. No significant difference in adiponectin levels was observed between CR and NCD mice (Supp Fig. 4a), which was in contrast to the pre-tumour levels (Fig. 2b). HFD mice displayed significantly reduced adiponectin compared to both CR and NCD controls post tumour (Supp Fig. 4a). When comparing serum levels pre- and post-tumour, adiponectin was significantly reduced post tumour for both CR and HFD groups, with levels in NCD mice remaining unchanged (Supp Fig. 4b). Leptin levels at tumour endpoint displayed a similar trend to pre-tumour endpoint levels, with a significant increase in leptin with HFD compared to CR and NCD (Supp Fig. 4c). When compared to pre-tumour endpoint, leptin levels were further increased at tumour endpoint for HFD mice (Supp Fig. 4d), likely reflecting the weight gain of HFD mice at this later timepoint (Fig. 4a). Serum levels of IGF-1 and insulin were also assessed at tumour endpoint. Compared to NCD mice, IGF-1 levels were decreased with CR, and increased with HFD (Supp Fig. 4e). Moreover, IGF-1 levels did not significantly differ from pre-tumour endpoint levels for any diet group (Supp Fig. 4f). Similar to IGF-1, insulin levels were significantly increased in HFD mice compared to both NCD and CR at tumour endpoint (Supp Fig. 4g). However, compared to pre-tumour endpoint, insulin levels were reduced at tumour endpoint in the HFD group likely reflecting long term HFD induced pancreatic beta cell dysfunction (Supp Fig. 4h).

## Discussion

Multiple myeloma remains an incurable blood cancer for which several risk factors for progression have been identified, including age, sex, race and obesity^36^. Despite obesity being one of the only known modifiable risk factors for myeloma, to date, no studies have investigated the potential of dietary intervention to delay myeloma progression. This study evaluated the impact of different dietary protocols (30% CR diet, NCD, and HFD) on the tumour microenvironment and subsequent development and progression of MM in the well characterised transplant Vk*MYC (Vk14451-GFP) C57BL/6J mouse model.

Feeding regimes in this study created altered metabolic and microenvironmental states, allowing the effect of various indices, including body weight, fat and lean mass, glucose metabolism, bone architecture, adipokine levels and haematological parameters, to be evaluated. CR mice displayed weight loss, improved glucose tolerance, decreased cortical bone, increased BM adiposity, increased anti-inflammatory adiponectin levels, and decreased pro-inflammatory IGF-1 levels, consistent with previous studies^27,29,37,38^. Of interest, despite the weight loss observed with CR, there was no significant change in fat, or fat mass relative to body weight in CR-fed mice, but an increase in lean mass relative to body weight. Given the increase in BMAT seen after 6 weeks of 30% CR, this result may reflect an underlying redistribution of fat, with decreased WAT and increased BMAT. Contrastingly, mice fed on a HFD for 12 weeks displayed weight gain, increased fat mass relative to body weight, reduced glucose tolerance, decreased trabecular thickness, and increased serum leptin levels, as previously described^39–42^. Whilst HFD increased whole body fat mass in our model, we did not observe an increase in BMAT, consistent with a previous study demonstrating that ≥ 16 weeks on HFD is required to increase BM adiposity^43^. In our model, 12 weeks of HFD was insufficient to alter serum adiponectin levels, however, serum adiponectin was significantly reduced at tumour endpoint (21 weeks of HFD feeding), highlighting the importance of HFD duration for the induction of certain obesity-induced metabolic indices. Overall, the dietary protocols used in this study lead to significantly altered microenvironmental states. Despite this, we observed no significant difference in BM tumour burden with either CR or HFD.

While no significant difference in BM tumour burden was observed with HFD, a significant increase in splenic tumour burden in HFD-fed mice was evident, with splenic tumour burden being highly correlated with spleen size. Interestingly, splenic but not BM tumour burden, was correlated with whole body tumour burden as measured by SPEP, suggesting that the spleen is a prominent site of MM tumour growth in this model. MM cells typically reside within hematopoietic niches and are thought to hijack normal signalling pathways to support their growth^44^. In mice, the red pulp of the spleen is a common site of extramedullary haematopoiesis^34,45^, and expansion of both red and white pulp is observed in diet-induced splenomegaly^46^. Given that the spleen is a hospitable site for Vk*MYC cells to reside, it is possible that the increased splenic tumour burden in HFD mice may not be due to changes in metabolic signalling but rather an increased availability of MM niches resulting from HFD-induced splenomegaly. Taken together, these results suggest that Vk*MYC cells may not be responsive to diet-induced microenvironmental changes.

While diet did not affect the BM tumour burden in this study, we cannot preclude the possibility that the duration of the feeding regimes may have been insufficient to observe a notable effect. A previous study, however, demonstrated that 5 weeks of HFD feeding prior to inoculation of 5TGM1 MM cells was sufficient to promote BM tumour growth in the non-permissive C57BL/6J mouse strain^16^. Interestingly, despite the ability of HFD-induced obesity to create a MM growth permissive environment, the authors found that HFD did not increase tumour burden relative to NCD-fed mice in the permissive KaLwRij mouse strain. Findings from our study, using the permissive Vk*MYC-C57BL/6J model, are in keeping with these results, suggesting that in mouse models of MM, diet-induced changes do not directly promote growth but instead create MM growth-permissive conditions.

The Vk*MYC (Vk14451-GFP) MM cells were initially generated by crossing mice which express GFP under the control of the gamma1 promoter^47^, with Vk*MYC mice that spontaneously develop MM due to an activating mutation in the proto-oncogene *MYC*^35^. Unlike other immunocompetent models of MM, such as the 5TGM1-KaLwRij model, the transplant Vk*MYC (Vk14551-GFP)/C57BL/6J MM model has the advantage of being relatively slow progressing with clinical features developing over ~ 9–12 weeks. Nevertheless, despite its slow progression, this model does not adequately reflect the MGUS to MM transition. In humans, the progression of MGUS to MM is relatively infrequent with only 1% of patients progressing each year^4^. Comparatively, the transplant Vk14451-GFP model of MM had 100% penetrance, with mice developing a disease that recapitulates the biological and clinical features of human MM. In patients, previous studies have identified the presence of *MYC* activation signatures in ~ 70% of newly diagnosed MM cases, with an absence in MGUS patients^48^. Similarly, studies analysing MYC protein levels by nuclear immunohistochemical staining found undetectable levels of MYC in the PCs of MGUS patients but expression in 84% of myeloma samples^49^. Population-based studies have associated high MYC protein expression with advanced MM and poor prognosis^50^. As such, constitutive *MYC* expression in Vk*MYC cells may not adequately reflect a pre-malignant MGUS state.

Interestingly, cancer cells with mutations in oncogenes, such as *MYC*, are less prone to adapt to fasting conditions, remaining highly proliferative. This phenomenon is utilised in fasting and chemotherapy adjuvant therapy whereby cancer cells are more resistant to dietary-induced changes to proliferation, while normal cells undergo cellular repair processes and slow growth. Differential proliferative responses to nutrient deprivation leads to improved chemotherapy targeting of cancer cells and a reduction in off-target side effects^51^. Therefore, whilst the Vk*MYC model may not be an ideal model to test the efficacy of a CR diet to delay the progression of MGUS to MM, further studies are still required to investigate the efficacy of fasting prior to chemotherapy to sensitise MM cells and improve patient outcomes.

While it is possible that hyperactivation of MYC promotes the growth of Vk*MYC MM cells irrespective of microenvironmental changes, it is also possible that the anti-tumorigenic effects of CR are being counteracted by pro-tumorigenic changes. In the BM, MM PCs interact closely with neighbouring cells which provide pro-tumorigenic factors to promote MM proliferation. Under CR, the BMME is densely populated with BM adipocytes. These adipocytes were hypothesised to be anti-tumorigenic, due to their change in phenotype and increased production of adiponectin under CR, however, these adipocytes may promote myeloma by acting as an energy source. Adipocytes are filled with lipid droplets which act as a reservoir of fatty acids that can be utilised in times of metabolic demand. Whilst BM adipocytes are more resistant to lipolysis than white adipocytes^52^, studies have shown that cancer cells are capable of modifying BM adipocyte phenotype^53^ and promoting lipolysis^54^. To this end, Panaroni et al. demonstrated that myeloma cells could induce adipocyte lipolysis and uptake fatty acids through fatty acid transporter proteins^55^. Moreover, recent studies have shown that MM cells are capable of downregulating BM-adipocyte secreted adiponectin by the production of TNF-α^56^. In line with this, results of our study found a significant decrease in serum adiponectin levels at tumour endpoint (Supp Fig. 4b). As such myeloma cells may be hijacking the BM adipocyte niche to promote their growth.

While it is well known that CR increases BM adiposity, recent studies have highlighted additional changes to BM cellularity in response to a CR diet, including a redistribution of immune cells to the BM and increased BM erythropoiesis. Studies by Contreras et al. and Collins et al. have shown that CR results in a loss of leukocytes in the periphery and a homing of memory lymphocytes to the BM^57–59^. In line with these findings, data from our study revealed decreased total white blood cells, neutrophils, lymphocytes and monocytes in the peripheral blood of CR mice (Fig. 3). The redistribution of immune subsets in CR is suggested to be an innate survival mechanism to protect important memory T cells during times of energy deficiency, and which can be reversed with refeeding. Collins et al. showed that the BM enhanced memory cell immune function during CR leading to better protection against secondary challenges, however the effect of this change on BM tumours remains unclear. Despite increased memory responses, primary immune responses against invading pathogens and tumour cells may be dampened due to hypocellularity in secondary lymphoid organs. In line with this, studies in other pathological contexts have shown reduced immune function in CR, with increased susceptibility to influenza A^60^ and bacteria^61^. Interestingly, Contreras et al. demonstrated that the homing of T cells to the BM under CR is driven by the CXCR4–CXCL12 axis. In MM, the CXCR4–CXCL12 axis plays a pivotal role in promoting the homing of MM cells to the BM due to the high expression of CXCR4 on MM PCs. As such, the immune cell redistribution occurring under CR may be inadvertently driving MM progression in the transplant Vk*MYC model by (a) reducing primary immune responses and (b) promoting MM cell BM homing.

Given the potential pro-tumorigenic effects of CR in the context of MM, it would be of interest to investigate the potential of other dietary intervention strategies such as intermittent fasting and alternative day fasting. These alternative forms of dietary intervention have not been reported to promote expansion of BMAT and changes to immune cell distribution but elicit many of CR’s beneficial effects. Moreover, these fasting strategies better mimic our ancestors’ diets, in which fasting was more common in times of food scarcity. Further work investigating the potential of dietary intervention would benefit from use of an alternative myeloma tumour model, in which tumour burden primarily resides in the BM and is responsive to microenvironmental changes. This would be more clinically relevant, given that MM is a human disease which predominantly resides in the skeleton, and interacts closely with neighbouring cells in the microenvironment.

Additional studies would benefit from understanding the mechanisms by which obesity drives MM, and how to reverse the pro-tumorigenic effects. Of interest, Lwin et al. found that switching C57BL/6J mice from a HFD to a NCD, 2 weeks post 5TGM1 MM cell inoculation, was sufficient to reduce whole body tumour burden as measured by serum paraprotein^16^. Given that these mice were maintained on a NCD for only 2 weeks prior to tumour endpoint, it is possible that diet may be directly driving MM proliferation rather than altering the microenvironment. Notably, inoculation of 5TGM1 MM cells into *ob/ob* mice, a genetically-induced model of obesity resulting from leptin deficiency, did not promote tumour growth^16^. Whilst this may suggest that leptin is a driving factor that promotes MM in obesity, it may also reflect an underlying importance of dietary composition in promoting MM progression. Notably, MM cells have been shown to uptake exogenous free fatty acids (FFAs)^55^, as such increased FFAs from increased dietary fat content may drive MM by altering cancer cell metabolism.

In summary, this study investigated the influence of diet (30% CR, NCD, or HFD) on tumour progression in the transplant Vk*MYC (Vk14451-GFP)-C57BL/6J pre-clinical model of MM. Results of this work demonstrated altered metabolic and microenvironmental states associated with dietary changes, however, dietary intervention was insufficient to affect BM tumour burden. Our findings suggest diet-induced BM changes may not be key drivers of MM progression in this model, however future studies are warranted to further understand the role of diet in MM disease progression.

## Methods

### Animal ethics

This study was approved by the South Australian Health and Medical Research Institute (SAHMRI) Animal Ethics Committee (ethics approval number: SAM21-016). Male C57BL/6J mice were bred and housed in pathogen free conditions at the SAHMRI Bioresources facility (SAHMRI, Adelaide, Australia). All experiments were performed in accordance to the Australian code for the care and use of animals for scientific purposes. Where applicable data and methods were described in line with the ARRIVE guidelines.

### Dietary regimes

From 4 weeks of age group-housed male C57BL/6J mice were randomly assigned either a high fat diet (HFD) (specialty feeds, Australia, SF16-096; 43% kcal fat), or normal chow diet (NCD) (specialty feeds, Australia, SF09-091, 23% kcal fat). At 9 weeks of age all C57BL/6J mice were transferred from group housing to single housing in fresh cages. From 9–10 weeks of age ad libitum food consumption of NCD mice was measured daily at 9:00 am. Mean food intake was calculated over the final 3 days to allow mice to adapt to the stress of single housing before assessing food consumption. From 10 weeks of age NCD mice were assigned to either remain ad libitum on a NCD or changed to a CR diet. Dietary assignment was based on groups with equal means and standard deviations for starting weights. Mice assigned to a CR diet received 70% mass of calculated normal chow ad libitum intake, equating to 2.4 g/day. The diet of CR mice was changed to specialty feeds SF21-016, a micronutrient enriched diet to prevent potential micronutrient deficiencies. The CR diet was administered once daily by placing patty pans of pre-weighed food directly into cages at 9:00 am. Mice were maintained on their assigned diet until humane endpoint at either 16 weeks of age (Pre-tumour Endpoint) or 25 weeks of age (Tumour Endpoint) (Fig. 1a). Comparison of nutritional parameters and dietary composition are shown in Supp Table 1.

### Glucose tolerance test

The morning of the glucose tolerance test (GTT), CR mice were provided a normal daily food portion at 6 am (2.4 g, SF21-016), while NCD and HFD mice remained ad libitum. At 8 am all mice were moved to new clean cages with food removed to begin a 6-h fast equivalent for all diet groups (08:00–14:00). At 2 pm fasting blood glucose levels were measured using a handheld glucometer (Accu-check, Roche, Australia). Mice were then administered 2 g/kg glucose by intraperitoneal injection with blood glucose readings taken at 15, 30, 60 and 120 min post injection. At fasting (0 min) and 30 min timepoints, whole blood samples were collected. Serum was isolated by centrifugation at 3000×*g* for 10 min and immediately frozen at − 80 °C for later assessment of insulin levels using a commercial insulin ELISA kit (EZRMI-13K, Millipore, MA, USA) as per manufacturer’s instructions. The GTT was performed 4 days prior to the pre-tumour endpoint to allow recovery of the mice prior to humane killing.

### Body composition

Body composition was measured at 16 weeks of age by EchoMRI body composition analyser (EchoMRI LLC, Houston, TX, USA) as per manufacturer’s instructions.

### Humane killing

At 5 pm on the day prior to termination mice were transferred to clean cages and provided with half their daily food intake to ensure equivalent fasting between diet groups. CR mice were provided with 1.2 g SF21-016, NCD mice were provided 1.7 g SF09-091, HFD mice were provided 1.7 g SF16-096. This approach was done to ensure any differences between groups reflect effects of longer-term CR, and not simply differences between fasted (CR) and non-fasted ad libitum mice. On the day of humane killing mice were anaesthetised by isoflurane inhalation and cardiac bled using a 26G needle followed by cervical dislocation.

### ELISA

Fasted blood samples were collected at the end of the study via cardiac puncture into Minicollect serum gel tubes (Cat# 450533, Greiner vacuette, Kremsmünster, Austria). Sera was isolated by centrifugation at 3000×*g* for 10 min at room temperature and stored in aliquots at − 80 °C. Commercial ELISA kits were used for the measurement of: leptin, adiponectin (EZML-82K and EZMADP respectively, Millipore, Burlington, MA, USA), and IGF-1 (MG100, R&DSystems, Minneapolis, USA) as per manufacturer’s instructions.

### Bone parameters

#### Bone isolation

Tibias were dissected from mice using scissors, razor blades and gauze to limit contamination by extraosseous tissue. Bones were fixed in 10% neutral buffered formalin (Ajax Fine Chem, NSW, Australia, cat#2518) for 24 h at 4 °C on a rocker then transferred to PBS at 4 °C.

#### Calcified bone μCT scanning

X-ray micro-computed tomography (μCT) was performed using a SkyScan 1276 (Bruker, Kontich, Belgium). Tibias were scanned at 60 kV/200 mA using a 0.25-mm aluminium filter, a 0.2 rotation step, and two-frame averaging with an isometric resolution of 5 μm/pixel. Images were reconstructed using NRecon software (Bruker) with a ring artefact reduction of 8, beam-hardening correction of 30%, and smoothing of 1.

#### Decalcification

Tibias were decalcified in a solution of 14% EDTA (E1644, Sigma-Aldrich, Burlington, MA, USA) pH 7.4 at 4 °C, with the EDTA solution replaced every 2–3 days. After approximately 2 weeks, decalcification was confirmed by X-ray imaging. Following this, bones were washed with PBS 3 times to ensure complete removal of the EDTA solution.

#### Osmium tetroxide staining

Tibias were stained with 1% osmium tetroxide solution [equal parts 2% osmium tetroxide (Electron Microscopy Sciences, Hatfield, PA, USA, cat#19192) and 0.1 M Sorensen’s buffer (19 mM KH_2_PO_4_, 81 mM Na_2_HPO_4_, pH 7.4)] as previously described^62,63^. The solution was left on the bones for 48 h then removed to a waste container containing oil for sufficient neutralisation. Bones were washed for a minimum of 72 h with buffer changed 2 × per day. All wash solutions were placed in waste container for decontamination.

#### Osmium tetroxide stained bone μCT scanning

Osmium tetroxide stained bones were scanned in a SkyScan 1276 (Bruker). Bones were scanned at 90 kV/200 mA using a 0.5-mm aluminium filter, a 0.2 rotation step, and two-frame averaging with an isometric resolution of 3 μm/pixel. Images were reconstructed using NRecon software (Bruker) with a ring artefact reduction of 8, beam-hardening correction of 30%, and smoothing of 1.

#### CTAn analysis

Analysis of the bone microarchitecture was performed using CTAn (Bruker). Trabecular and cortical regions of interest (ROI) were selected for analysis of calcified tibias. For analysis of proximal and midshaft regions, areas of 400 microtomographic slices were selected 60 and 1200 slices distal to the growth plate respectively. For osmium tetroxide stained samples, BMAT quantification was performed on whole bone, as well as proximal and distal regions with the boundary defined by the tibia-fibula junction. In all cases, regions of interest (ROI) were manually drawn and analysed in CTAn using adaptive thresholding.

### Vk*MYC model of MM

At 16 weeks of age C57BL/6J mice were inoculated with Vk14451-GFP whole BM suspensions kindly provided by Dr Michelle McDonald (Garvan Institute, NSW, Australia). Cell stocks were expanded by serial passage in vivo through BM. P3 cell stocks were thawed into RPMI-1640 containing 10% fetal calf serum (FCS) and subsequently resuspended in sterile phosphate buffered saline (PBS) equivalent to 2.5 × 10^5^ GFP+ tumour cells/mL calculated based on the percentage of GFP+ cells by flow cytometry. Mice were injected intravenously with 100 μL of cell suspension equating to 25,000 MM PCs. MM tumour was allowed to develop until ethical endpoint was reached. Tumour burden was monitored weekly by serum protein electrophoresis (SPEP) on a Sebia Hydragel b1/b2 kit (Sebia, Norcross, GA, USA) as previously described^64^. Hind limbs (femurs/tibias) and spleens were dissected at endpoint for subsequent flow cytometric or morphological analysis. Two mice with missed intravenous injections were excluded from analysis.

### Flow cytometry

For analysis of MM PC within the BM, femurs and tibias were crushed in PFE buffer [PBS, 2% FCS, 2 mM ethylenediamine tetra acetic acid (EDTA)] using a mortar and pestle. The resulting BM cell suspension was homogenised and passed through a 70 μm filter. For analysis of MM PC within the spleen, spleens were excised, cleaned of connective tissue and homogenised between two frosted histology slides. Splenocytes were subsequently washed with PFE, filtered, and subjected to 1 × round of red blood cell (RBC) lysis. Spleen and BM samples were resuspended in PFE and immediately run on the BD LSRFortessa™ X20 with subsequent analysis performed using FlowJo v10.0.8 software. In all instances, a BM from a naïve (non-injected) mouse was used as a negative control for gating.

### qPCR

Total RNA was extracted from BM cells and splenocytes using TRIzol reagent (Invitrogen, MA, USA) and isopropanol precipitation according to manufacturer’s recommendations. cDNA was synthesised from 1.5 μg total RNA using Superscript™ IV reverse transcriptase (Invitrogen) as per manufacturer’s protocol. qPCR was performed on the Quantstudio 3 Real-Time PCR System (Applied Biosystems, MA, USA) using RT^2^ SYBR^®^ Green reagent (QIAGEN, Hilden, Germany). Gene expression was analysed using the ΔCt method (2^−ΔCt^) normalised to *RPLP0*. *RPLP0* normalisation was chosen based on its known stability as a reference gene in HFD-fed C57BL/6J mice^65^, and its stability as a housekeeping gene in BM^66^. The following primers were used: *RPLP0* F: 5′ AGATTCGGGATATGCTGTTGGC 3′, *RPLP0* R: 5′ TCGGGTCCTAGACCAGTGTTC 3′, *TNFα* F: 5′ CCTGTAGCCCACGTCGTAG 3′, *TNFα* R: 5′ GGGAGTAGACAAGGTACAACCC 3′, *IL1β* F: 5′ GCCACCTTTTGACAGTGATGAG 3′, *IL1β* R: 5′ AGCTTCTCCACAGCCACAAT 3′, *IL6* F: 5′ TAGTCCTTCCTACCCCAATTTCC 3′, *IL6* R: 5′ TTGGTCCTTAGCCACTCCTTC 3′, *MPO* F: 5′ TCCCACTCAGCAAGGTCTT 3′, *MPO* R: 5′ TAAGAGCAGGCAAATCCAG 3′, human *MYC* F: 5′ CGTCCTCGGATTCTCTGCTC 3′, human *MYC* R: 5′ GCTGCGTAGTTGTGCTGATG 3′.

### Morphological analysis

Spleens were excised from tumour-bearing C57BL/6J mice at experimental endpoint and visually inspected for the presence of extramedullary tumour lesions. Spleens were photographed by a ruler for later assessment of spleen length to the closest millimetre by ImageJ analysis software.

### Peripheral blood counts

Whole blood samples were collected from mice by terminal cardiac bleed into Minicollect K3 EDTA tubes (Cat# 450531, Greiner vacuette, Kremsmünster, Austria). Complete blood counts were performed using a HEMAVET950 automated blood analyser (Drew Scientific, FL, USA), according to the manufacturer’s instructions.

### Statistical analysis

All statistical analyses were performed using GraphPad PRISM (version 9.00; GraphPad Software, La Jola, CA, USA). Groups were compared using one-way or two-way analysis of variance (ANOVA) with Tukey’s or Sidak’s multiple comparisons post-tests, as indicated. Correlation was assessed using Pearson’s correlation coefficient.

## Supplementary Information

Supplementary Information.

## Data Availability

All data generated during this study are included in this published article (and its Supplementary Information files).
