# Supplementary material for: Calorie restriction has no effect on bone marrow tumour burden in a Vk*MYC transplant model of multiple myeloma
Source: Sci Rep. 2022 Jul 30;12:13128. doi: 10.1038/s41598-022-17403-9 (PMC9338941; doi:10.1038/s41598-022-17403-9)
Supplement: Supplementary file 1 — Supplementary Information. [file 41598_2022_17403_MOESM1_ESM.pdf]

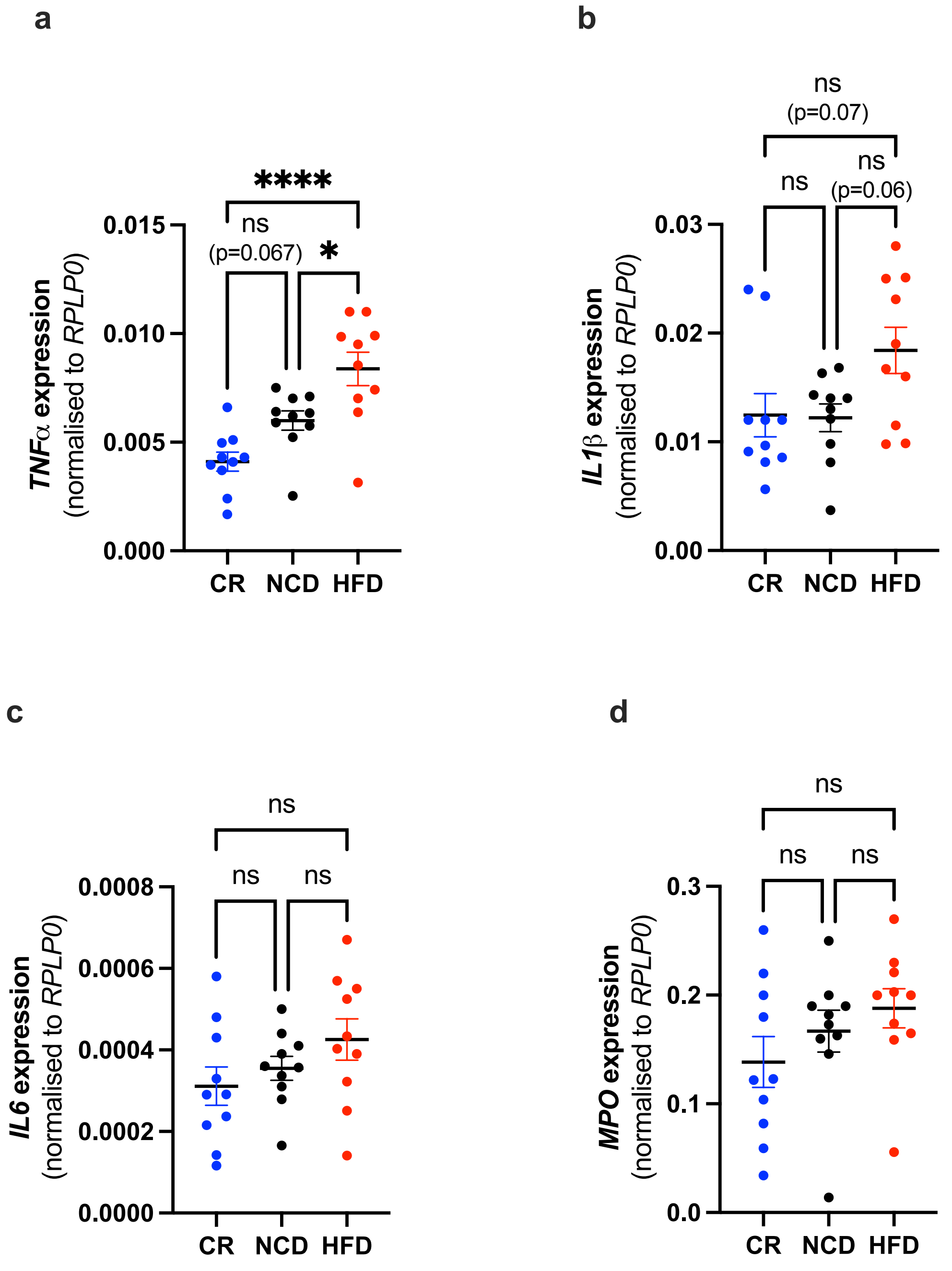

Supplementary Figure 1. Gene expression of inflammatory markers in tibial BM in response to diet. Pre-tumour endpoint BM gene expression of a) *TNFα*, b) *IL1β*, c) *IL6*, d) *MPO*. Error bars SEM, n=10/group, ns p>0.05, * p<0.05, ** p<0.01, *** p<0.001, **** p<0.0001, ordinary one-way ANOVA with Tukey's multiple comparisons test

a

b

c

d

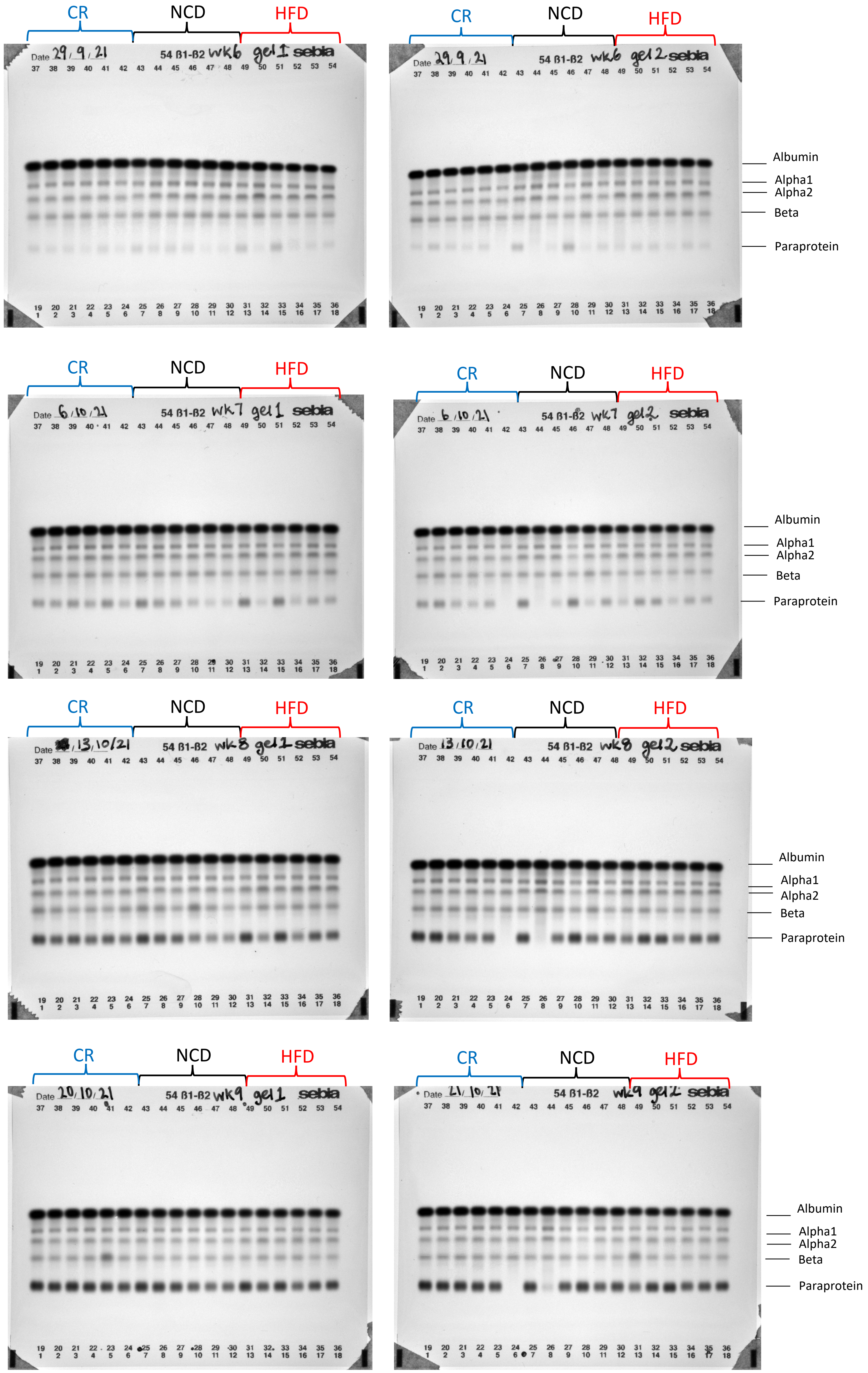

Supplementary Figure 2. Serum paraprotein gel electrophoresis (SPEP) gel images. Relative intensity of serum paraprotein bands was analysed at a) week 6 post tumour initiation, b) week 7 post tumour initiation, c) week 8 post tumour initiation, and d) week 9 post tumour initiation. At each timepoint, serum from n=12 mice/group was processed in parallel with serum from n=6 mice/group separated and analysed on each gel. In all cases lanes 1-6 represent samples from CR-fed mice, lanes 7-12 represent samples from NCD-fed mice, and lanes 13-18 represent HFD-fed mice.

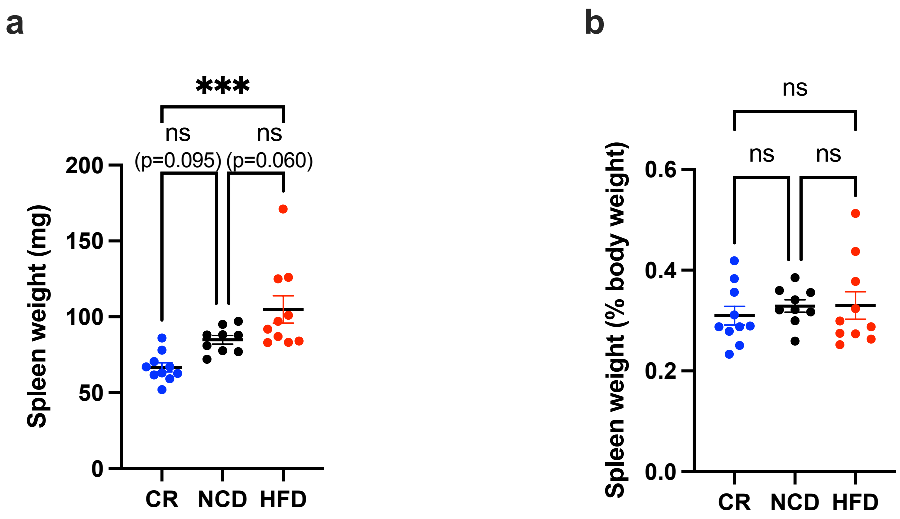

Supplementary Figure 3. Diet affects spleen size at pre-tumour endpoint. a) Spleen mass, b) Spleen mass (% body weight). Error bars SEM, data n=9-10/group, ns p>0.05, * p<0.05, ** p<0.01, *** p<0.001, **** p<0.0001, ordinary one-way ANOVA with Tukey's multiple comparisons test

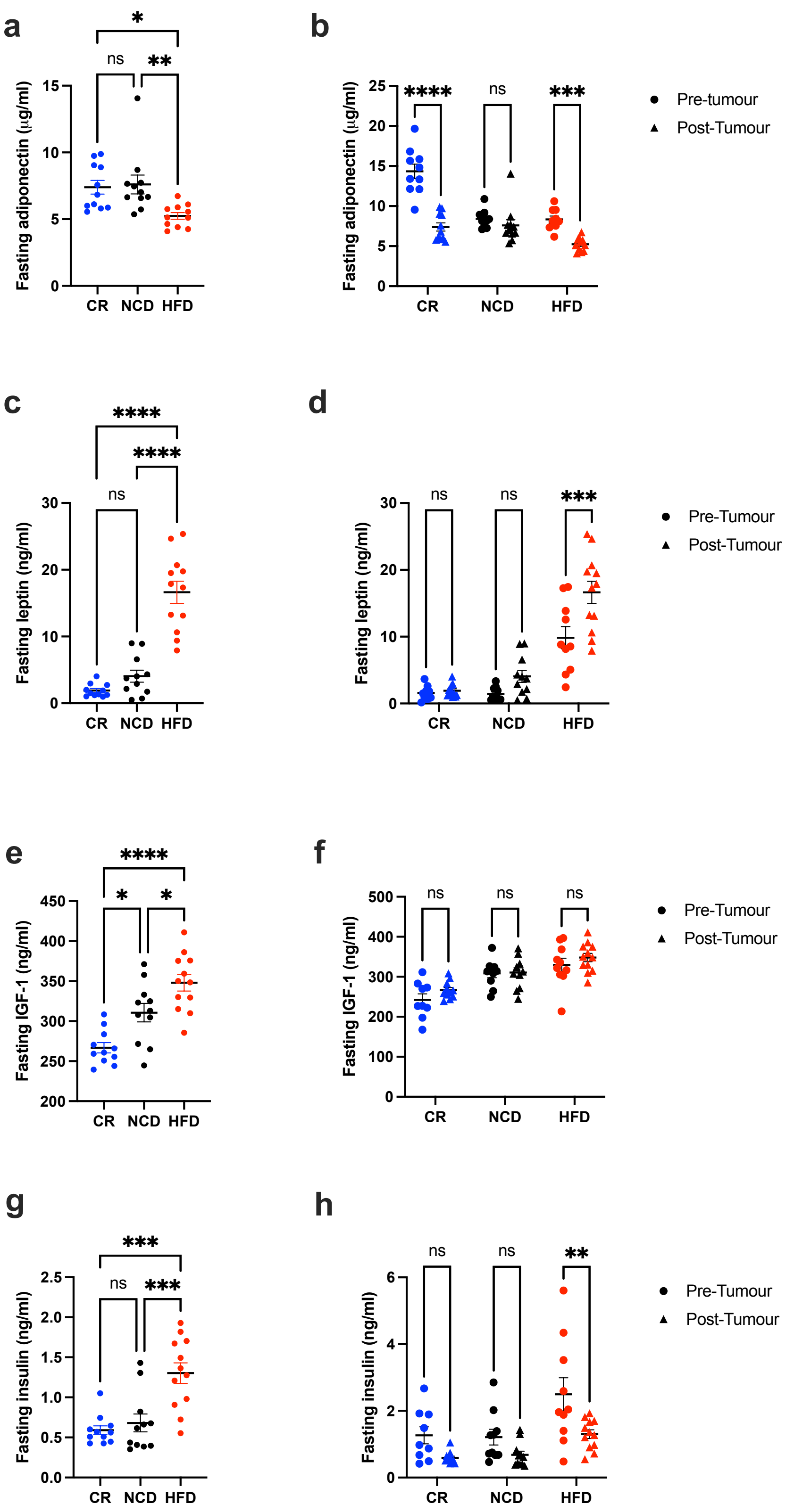

Supplementary Figure 4. Serum adipokine and cytokine levels at pre- and post-tumour endpoints. Tumour endpoint serum levels, and comparison to pre-tumour endpoint levels for a, b) Adiponectin, c, d) Leptin, e, f) IGF-1, and g, h) Insulin. Error bars SEM, (a, c, e, g) n=11-12 mice/group, Ordinary one-way ANOVA with Tukey's multiple comparisons test, (b, d, f, h) n=9-12 mice/group, two-way ANOVA with Sidak's multiple comparisons test, ns $p>0.05$, * $p<0.05$, ** $p<0.01$, *** $p<0.001$, **** $p<0.0001$.

Supplementary Table 1. Composition of Experimental Diets

	CR (SF21-016)	NCD (SF09-091)	HFD (SF16-096)
**Calculated Nutritional Parameters as Fed**			
Protein	19.30%	19.40%	19.70%
Total Fat	6.90%	7.00%	23.00%
Crude Fibre	4.60%	4.70%	4.70%
AD Fibre	4.60%	4.70%	4.70%
Digestible Energy	15.9 MJ / Kg	16.1 MJ / Kg	19.7 MJ / Kg
% Total calculated digestible energy from lipids	15.80%	16.00%	43.40%
% Total calculated digestible energy from protein	20.00%	21.00%	17.30%
**Ingredients**			
Casein (Acid)	196 g/Kg	200 g/Kg	200 g/Kg
Sucrose	98 g/Kg	100 g/Kg	418 g/Kg
Canola Oil	69 g/Kg	70 g/Kg	50 g/Kg
L Methionine	3.0 g/Kg	3.0 g/Kg	3.0 g/Kg
Calcium Carbonate	18.7 g/Kg	13.1 g/Kg	13.1 g/Kg
Sodium Chloride	3.7 g/Kg	2.6 g/Kg	2.6 g/Kg
AIN93 Trace Minerals	2.0 g/Kg	1.4 g/Kg	1.4 g/Kg
Potassium Citrate	3.5 g/Kg	2.5 g/Kg	2.5 g/Kg
Potassium Dihydrogen Phosphate	9.8 g/Kg	6.9 g/Kg	6.9 g/Kg
Potassium Sulphate	2.3 g/Kg	1.6 g/Kg	1.6 g/Kg
Choline Chloride (75%)	3.6 g/Kg	2.5 g/Kg	2.5 g/Kg
AIN93 Vitamins	21.4 g/Kg	15 g/Kg	15 g/Kg
Vitamin K Supplement	1.2 g/Kg	0.87 g/Kg	0.87g / Kg
Cellulose	49 g/Kg	50 g/Kg	50 g/Kg
Wheat Starch	390 g/Kg	399 g/Kg	-
Dextrinised Starch	129 g/Kg	132 g/Kg	-
Pregelled Wheat Starch	-	-	50 g/Kg
Cholesterol USP	-	-	1.9 g/Kg
Cocoa Butter	-	-	50 g/Kg
Hydrogenated Vegetable Oil (Copha)	-	-	131 g/Kg
